# The impact of middle managers’ digital leadership on employee work engagement

**DOI:** 10.3389/fpsyg.2024.1368442

**Published:** 2024-03-28

**Authors:** Zhenli Li, Cuibai Yang, Zhuohang Yang, Yunlu Zhao

**Affiliations:** ^1^School of International Studies, Sichuan University, Chengdu, China; ^2^School of Law, Sichuan University, Chengdu, China; ^3^Chuan Neng (Hainan) International Industry and Commerce Co., Ltd., Haikou, China

**Keywords:** middle management, digital leadership, work engagement, employee empowerment, affective commitment, emotional intelligence

## Abstract

**Background:**

In the rapidly evolving digital landscape, the role of middle managers in organizational structures and processes is increasingly pivotal. Positioned at the nexus of strategic directives and operational execution, they play an important role in driving digital transformation. This study discusses the under examined domain of middle managers’ digital leadership and its impact on employee work engagement in the context of digital transformation.

**Design:**

Drawing on Social Exchange Theory, this study investigates the influence of middle managers’ digital leadership on employee work engagement through the analysis of survey data from 559 respondents across 11 listed companies in Southwest China. It examines the roles of employee empowerment and affective commitment as pivotal mediating variables and investigates the moderating effect of emotional intelligence in these relationships.

**Research purposes:**

The study aims to elucidate the mechanisms by which middle managers’ digital leadership fosters employee work engagement, highlighting the importance of emotional intelligence, empowerment, and affective commitment in this process.

**Findings:**

The study reveals that middle managers’ digital leadership has a significant positive impact on employee work engagement. Employee empowerment and affective commitment serve as mediating factors in the relationship between middle managers’ digital leadership and employee work engagement. Emotional intelligence moderates the effect of middle managers’ digital leadership on employee empowerment. Meanwhile, emotional intelligence further moderates the chain mediating of employee empowerment and affective commitment between middle managers’ digital leadership and employees’ work engagement.

**Implications:**

This research offers valuable insights into the dynamics of leadership and engagement in the digital era, emphasizing the need for organizations to foster digital leadership capabilities in middle management. It provides practical implications for enhancing employee work engagement through strategic digital leadership, emphasizing the role of employee empowerment, affective commitment and emotional intelligence in adapting to digital transformation.

## 1 Introduction

The surging advances of digital technologies have revolutionized organizational structures and processes, prompting an imperative reexamination of leadership roles and styles in contemporary enterprises (Bäck et al., 2020). This transformation is acutely exemplified in the pivotal role of middle managers, who find themselves at the confluence of strategic planning and operational execution. As key agents of change, middle managers bridge the gap between the highest strategic decision-making levels and practical actual execution. They wear multiple hats – from interpreters and integrators of plans to innovators and relationship coordinators (Harding et al., 2014). The advent and integration of digital technologies have transformed how middle managers communicate and disseminate strategies from top-level management and engage employees at the operational level, thereby reshaping hierarchical dynamics and control within organizations (Avolio et al., 2014). Such changes underscore the need to enhance digital leadership capabilities among middle managers. Digital leadership transcends mere technical proficiency; it encompasses a broader array of skills, including strategic thinking, adaptability, and the capability to inspire and guide teams through the complex landscape of digital transformation (Tigre et al., 2023). Digital leadership, especially within the realm of middle management, transcends mere technical proficiency. It embodies a unique blend of capabilities essential for navigating the digital era (Van Doorn et al., 2023). Key among these is strategic thinking, enabling middle managers to align digital initiatives with broader organizational goals. Adaptability is another crucial trait, allowing them to respond swiftly and effectively to the ever-changing digital landscape. Furthermore, this form of leadership emphasizes the ability to inspire and guide teams, fostering a culture of innovation and collaboration essential for digital transformation (Koponen et al., 2023). Beyond affecting managerial roles, the digital revolution has profound implications for employee work engagement (Juyumaya and Torres, 2023). Digital technologies alter the work environment, task structures, and communication channels, leading to shifts in employee roles, expectations, and engagement levels (Toth et al., 2020).

In this evolving digital landscape, employee engagement becomes a critical metric of an organization’s ability to adapt and thrive. Employee work engagement, characterized by high levels of vigor, dedication, and commitment that employees show at work, is a critical determinant of organizational success (Kahn, 1990). Engaged employees demonstrate higher productivity, innovation, and commitment, contributing significantly to organizational resilience and adaptability in times of digital transformation (Oberländer and Bipp, 2022). However, the existing body of literature has predominantly concentrated on conceptualizing digital leadership at senior management levels (Avolio et al., 2014), frequently overlooking the nuanced and multifaceted role of middle managers in digital contexts, especially in terms of how they can guide their employees to enhance work engagement. The proliferation of digital technologies in the workplace has profoundly altered the landscape of employee work engagement. While these technologies offer innovative platforms for collaboration and efficiency, they also bring unique challenges that can impact employee work engagement. The digital work environment often leads to blurred boundaries between work and personal life, potentially leading to burnout. In this context, the role of middle managers becomes crucial in mitigating the potential negative impacts of digitalization on employee work engagement. While existing research underscores the positive impact of digital leadership on employee engagement, there is a significant gap in studies focusing specifically on the role of middle managers in this context (Busse and Weidner, 2020). This gap highlights the critical need for in-depth exploration into how middle managers’ digital leadership influences employee engagement, considering their unique position within organizational hierarchies and the distinct challenges and opportunities they encounter during the digital transformation journey (Wang et al., 2021; Klebe et al., 2023). This study aims to fill this gap by examining the relationship between middle managers’ digital leadership and employee work engagement. This study addresses the following questions: (1) Does the middle managers’ digital leadership bolster employee work engagement? (2) How does middle manager’s digital leadership influence employee work engagement.

In summary, this study endeavors to fill the gap in existing research by examining the role of middle managers’ digital leadership in influencing employee work engagement. By adopting a Social Exchange Theory perspective, the research provides new insights into the dynamics of leadership and work engagement within the digital era. The findings of this study are expected to offer valuable insights for both theory and practice, particularly in enhancing our understanding of the dynamics of digital leadership at the middle management level and developing strategies to foster higher employee work engagement in digitally transforming organizations.

## 2 Theoretical background

### 2.1 Middle managers’ digital leadership

Middle managers occupy a unique position in organizational hierarchies, serving as the link between senior leadership and the operational core (Funke et al., 2023). They are tasked with overseeing business units at the intermediary levels of the organization, nestled between the senior echelons of strategic management and the basic supervisory layer. Distinct from both senior executives and rank-and-file staff, middle managers embody roles that are multifaceted and complex (Yang and Leposky, 2022). They wield control over their subordinates, yet simultaneously work under the directives of higher management, positioning them as figures who are both controller and controlled. Concurrently, middle managers navigate a delicate balance, resisting certain directives from senior management while confronting resistance from their own teams, thus playing the dual roles of resistor and resisted. In the sphere of corporate digitalization, while senior managers set strategic directions, it is the middle managers who are instrumental in grounding and actualizing organizational change, which is key to realizing a company’s digital transformation (Putra et al., 2023). Specifically, senior managers often define change initiatives in broad, visionary terms, focusing on the organization’s overall value chain but frequently lacking in detailed execution strategies. Middle managers, situated at the nexus of the organization’s critical knowledge flow, possess the unique ability to amalgamate and internalize vital inputs from both strategic and operational levels, translating high-level strategies into solid, actionable steps (Hassard and Morris, 2022). Moreover, middle managers play a pivotal role in aligning the digital transformation strategy with the everyday tasks of their teams, seamlessly integrating routine operational activities with overarching digital objectives. Their proximity to the workforce and frequent interactions with employees position them effectively to identify, understand, and address employee concerns, thereby mitigating change resistance and fostering positive work attitudes and behaviors (Baptista et al., 2020). Despite the recognition of middle managers’ vital role in organizational change and innovation, there is a notable gap in research focusing on their contribution to the digitalization journey of enterprises.

Digital leadership, a critical element in the digital transformation of enterprises, is characterized as an influential process that operates in a direct and broader context, mediated by digital technology (Avolio et al., 2014). This leadership style catalyzes change at individual, team, and organizational levels, encompassing attitudes, emotions, thought processes, behaviors, and performance outcomes. Digital leadership is marked by its change-driven nature, commitment to tasks associated with digital twins, alignment with the cutting edge of digital advancements, visionary outlook, and strong adaptability (Weber et al., 2022). As a cornerstone of digital transformation in enterprises, digital leadership accentuates interaction among organizational stakeholders, steering followers to acquire, leverage, and innovate with digital technologies for value creation in the digital domain, thereby propelling successful digital transformation. Digital leadership, therefore, places a significant emphasis on building managerial competencies in areas like digital communication, socialization, change management, team dynamics, technological proficiency, and fostering digital trust (Tuerk, 2023). Existing research predominantly revolves around defining digital leadership, outlining its characteristics, developing related competencies, and exploring its impact on corporate digital transformation from a higher-order theoretical perspective, such as enhancing digital platform capabilities and breakthrough innovation (Abbu et al., 2022). Nevertheless, there’s a conspicuous scarcity of research delving into the nuanced impacts of digital leadership at a micro-level, particularly concerning employee cognition, emotional and in particular on middle managers who are structurally proximate to the employee base.

### 2.2 Employee work engagement

Work engagement represents an individual’s active participation in his or her professional role, characterized by an employee’s commitment of extensive cognitive, emotional, and physical resources to his or her work (Kahn, 1990). This engagement is fundamentally influenced by three psychological conditions arising from the work role: the perception of meaningfulness in one’s job, a sense of security, and the accessibility of necessary resources. The fulfillment of these conditions is crucial in determining whether employees will invest themselves fully in their work roles. Leadership, as a pivotal organizational element, significantly impacts employee work engagement. Employees exhibiting high levels of engagement typically show increased willingness, enthusiasm, and vigor in their work processes, demonstrating a proactive stance in adapting to the changes ushered in by organizational transformations. They immerse themselves with fervor in transformation activities, contributing to elevated job performance, enhanced innovative capabilities, and a rise in organizational citizenship behaviors (Albrecht, 2012). In the context of the uncertainties brought about by digital transformation, employees operating in digital work settings experience their roles primarily through a digital presence, which in turn influences their degree of work engagement. Research has shown that, in a digital work environment, besides the immediate causes such as team fragmentation, lack of social interaction, social support, and individual characteristics like self-discipline, the digital milieu that employees are part of — particularly the leadership behavior exhibited in digital processes — is a critical predictor of work engagement (Pass and Ridgway, 2022). Despite extensive research on digital leadership and employee work engagement by scholars around the world, there are still some gaps in these studies (Busse and Weidner, 2020; Maheshwari et al., 2024). These gaps are primarily evident in two domains. First, most researches, based on high-level theories, focuses on the intricacies and impacts of senior management’s digital leadership, with insufficient exploration into the domain of middle management’s digital leadership. Especially in digital contexts, middle managers need to enhance their digital leadership capabilities to cope with changes in the leadership environment and to connect with followers emerging in the digital era. Middle managers play an essential role in bridging the gap between employees and top-level strategic management. They meet employee needs, tweak incentive mechanisms, overcome career barriers, and identify set, thus motivating employees to adapt proactively and engage wholeheartedly in the digitalization process. Second, the influence of middle management’s digital leadership on employee work engagement deserves further exploration. While existing studies, based on Social Exchange Theory, have acknowledged the significant positive impact of digital leadership on work engagement and the mediating role of perceived organizational support, there’s a notable absence of investigation into the social interaction dynamics between middle managers and employees.

### 2.3 Social exchange theory

Social Exchange Theory (SET) offers a nuanced understanding of social behavior through the prism of reciprocal exchange (Surma, 2016). This theory suggests that social interactions are essentially transactions driven by an evaluation of rewards and costs. In both personal and professional domains, relationships are sustained and nurtured through a series of reciprocal actions, where individuals strategically aim to maximize benefits and minimize drawbacks (Widegren, 1997). The concept of reciprocity is at the heart of SET, which posits that interactions are motivated by the expectation of future returns or benefits, thus fostering and reinforcing relational bonds over time.

Rooted in Social Exchange Theory, there emerges a clear understanding that managers, particularly those in positions of structural advantage with access to resources, play a crucial role in empowering their employees (Baird and Wang, 2010). This employee empowerment, achieved through a blend of structured organizational practices and informal support mechanisms, not only improves the employees’ work environment but also significantly boosts their performance. The interaction between managers and employees is a dance of mutual exchange and commitment. Employees tend to deepen their engagement in anticipation of receiving greater support and empowerment from their managers (Ouerdian et al., 2021). Affective commitment, serving as a barometer of loyalty, reflects an employee’s sense of identity, involvement, and emotional bonding with the organization. This aspect is particularly pronounced in cultures that value interpersonal relationships, such as in China, where affective commitment is often expressed through unwavering loyalty and dedication to leaders (Robert and Vandenberghe, 2021). This commitment can reciprocally lead to achieve work conditions and mutual enhancement.

The impact of employee empowerment and affective commitment goes beyond mere engagement; they shape how employees perceive and evaluate middle managers’ digital leadership, thus are key prerequisites for work engagement. SET also sheds light on how individual differences influence the nature and outcomes of social exchanges. In the digital age, interactions between middle managers and employees predominantly take place through digital channels, leading to a reduction or loss of vital emotional cues — a challenge that affects emotional comprehension and response (Avolio et al., 2014). This shift underscores the importance of including emotional intelligence, an individual characteristic, in interpreting and responding to managerial cues. Consequently, this study, anchored in Social Exchange Theory, explores the intricate exchange relationship between middle managers and employees. Using employee empowerment and affective commitment as mediating variables and emotional intelligence as a moderating factor, the study empirically investigated the mechanisms and boundary conditions under which middle managers’ digital leadership affects employees’ work engagement.

## 3 Research hypotheses

### 3.1 Middle managers’ digital leadership and employee work engagement

Work engagement is conceptualized as an intricate process where employees shape their roles through the dedicated engagement of physical, cognitive, and emotional resources in their work-related activities. This process not only facilitates the application and expression of self in the professional domain but also acts as a catalyst for superior work performance and enriched interpersonal connections (Kahn, 1990). Employees’ decision to engage in work roles is contingent upon the fulfillment of a triad of psychological states derived from these roles: the sense of psychological meaningfulness, safety, and availability. Psychological meaningfulness underscores the perceived return on investment in work roles, psychological safety addresses the impact of the work environment on role enactment, and psychological availability pertains to the accessibility of personal resources for the employee. Leadership behavior, as evidenced in contemporary research, emerges as a critical organizational factor in influencing employee work engagement (Rahmadani and Schaufeli, 2022). Middle managers, positioned strategically within the organizational value chain, play a pivotal role in this context. Their digital leadership is instrumental in translating overarching digital transformation strategies into actionable, frontline behaviors (Linder and Torp, 2017; Verma and Garg, 2024). This leadership style equips employees with a refreshed cognitive framework, enabling them to appreciate the imperative and advantages of digital transformation, thereby intensifying their sense of psychological meaningfulness associated with their work roles. Moreover, given their crucial role in the conduct of organizational information, middle managers focus their digital leadership on synthesizing strategic directives from senior management with solid operational data. This synthesis provides a clearer vision of digital transformation (Birken et al., 2012), assisting employees in overcoming apprehensions related to the transition, thus bolstering their psychological safety within their work roles. In addition, their proximity to the workforce in the organizational hierarchy enables middle managers to tune into the employees’ evolving psychological states during the digital transformation. By providing targeted resources and support tailored to the needs of digital transformation (Heyden et al., 2017), they enhance employees’ perceptions of psychological availability in relation to their work roles. Viewed through the lens of Social Exchange Theory, when middle managers utilize digital technologies to assess employee capabilities and alleviate the physical and psychological strains associated with work roles, employees are likely to reciprocate by elevating their level of work engagement. This reciprocal dynamic fosters the evolution of the social exchange relationship with middle managers. In light of these considerations, the following hypothesis is proposed:

*H1*: Middle managers’ digital leadership has a significant positive impact on employee work engagement.

### 3.2 Middle managers’ digital leadership, employee empowerment and work engagement

Employee empowerment refers to an employee’s recognition of their capacity, authority, autonomy, and the skills and resources necessary to navigate their work environment (Men, 2011). According to Social Exchange Theory, empowerment is a relational concept, that focuses on the delegation and sharing of power during interactions between managers and employees (Conger and Kanungo, 1988). In the dynamics between managers and employees, empowerment is influenced through three key mechanisms: power-sharing, motivational support, and developmental support (Udod et al., 2020). Power-sharing manifests in the form of information sharing and participation in decision-making, motivational support is reflected in encouraging autonomy, expectation management, efficacy support, rewards, while developmental support is embodied through modeling and guidance. During the process of digital transformation, middle managers, also recipients of the digital transformation strategy, increasingly require support from employees. Therefore, their digital leadership emphasizes employee participation and cooperation, encouraging employees to express ideas and opinions, and fostering the flow, updating, and iteration of digital transformation-related information at the operational level by building shared information pools (Tigre et al., 2023). This enhances the quality and quantity of information employees receive and increases their opportunities for decision-making participation.

Moreover, middle managers are closer to their employees in the organizational structure and have a clearer understanding of their skills and strengths. Thus, digital leadership at this level focuses on transitioning from directive to participatory management. Increased decision-making involvement in the digital transformation process leads employees to believe in their capacity to contribute (Van Wart et al., 2017). Unlike the abstract, cutting-edge digital visions painted by senior managers, middle managers’ digital leadership excels in aligning digital strategies with specific teams and individuals, thereby translating them into actionable plans. By encouraging self-management and self-leadership among employees, this approach fosters participation in digital transformation, conveying a message of appreciation and trust from middle managers to their employees (De Waal et al., 2016). Additionally, middle managers’ digital leadership emphasizes continual learning, application, and innovation of new knowledge related to digital technologies, providing guidance and training to employees during the digital transformation, thereby further elevating their perception of empowerment (Liu et al., 2020). Therefore, this study posits that the higher the level of middle managers’ digital leadership, the more likely it is to enhance employees’ perception of empowerment.

Employee empowerment, as a mechanism influenced by organizational policies, human resource practices, and social network structures, helps to unleash employees’ inner potential and enrich their psychological resources, thereby improving their work state. First, middle managers’ digital leadership, by respecting, valuing, and appreciating employees’ participation in the digital process (Heyden et al., 2017), leads employees to perceive their work roles as valuable and meaningful, creating a heightened sense of psychological meaningfulness. Second, a higher perception of empowerment imbues employees with a stronger sense of power, control, and influence within the organization, alleviating fears and concerns about negative outcomes in their work roles and fostering a robust sense of psychological safety (Weber et al., 2022). Furthermore, with the aid of middle managers’ digital leadership, employees enhance their perception of their capabilities, organizational status, and autonomy as they gain decision-making rights and access to digital information resources (Monje-Amor et al., 2021). Social Exchange Theory posits that power reflects an individual’s ability to obtain resource in return. Consequently, the empowering actions of middle managers’ digital leadership facilitate employees in improving their resource return capabilities, making it more likely for employees to reciprocate by increasing their cognitive, emotional, and physical resource investments in their work roles. Based on this analysis, the following hypothesis is proposed:

*H2*: Employee empowerment mediates the significant positive impact of middle managers’ digital leadership on employee work engagement.

### 3.3 Middle managers’ digital leadership, affective commitment, and work engagement

Affective commitment refers to an employee’s psychological identification, emotional attachment, and involvement with their organization. It is characterized by a deep-rooted desire of employees to contribute to the organization’s success, influenced by the need to sustain a reciprocal social exchange relationship, shared values, and a sense of emotional belonging (Liu et al., 2011). According to Social Exchange Theory, the intensity of affective commitment is correlated with the degree to which managers acknowledge and care about their employees’ contributions. Middle managers, pivotal in the implementation of digital transformation, face the dual role of both executing and experiencing this organizational change. Consequently, their digital leadership is more attentive to analyzing emotional changes in employees, accurately identifying their responses to digital transformation, and taking targeted actions to address or collaboratively resolve related challenges (Vandenberghe, 2021). This approach helps to fulfill employees’ needs for emotional support, belonging, and respect. Moreover, middle managers’ digital leadership extends beyond valuing individual employee growth and development during digital transformation; it also emphasizes fostering digital team dynamics. Constructive social exchange relationships are nurtured through bidirectional and frequent digital interactions in the digital environment. By guiding employees in developing shared values, positive emotions, and a sense of duty are fostered, encouraging them to proactively form emotional connections with the organization based on reciprocal norms (Shao et al., 2022). Thus, this study suggests that the higher the level of middle managers’ digital leadership, the greater the enhancement of employees’ affective commitment. Affective commitment emotionally aligns employees with organizational goals and responsibilities, motivating them to improve their work attitudes and behaviors toward achieving these goals. First, employees with higher affective commitment are more likely to embrace and feel emotionally connected to the goals of digital transformation, thereby increasing the psychological significance of their roles in this process and consequently, their willingness to enhance work engagement (Morin et al., 2016). Second, employees with strong affective commitment typically exhibit higher resilience and adaptability, greater job satisfaction, and a willingness to invest more time and effort in their work, resulting in elevated levels of work engagement. Furthermore, employees not only have a clearer understanding of the necessity and value of digital transformation, but also combine it with personal value realization under the influence of digital leadership of middle managers. This leads to a stronger inclination to intertwine the organization’s future and development with their personal value pursuits (Pentareddy and Suganthi, 2015), prompting them to invest more resources in their work roles. Based on this analysis, the following hypothesis is proposed:

*H3*: Affective commitment mediates the significant positive impact of middle managers’ digital leadership on employee work engagement.

### 3.4 Middle managers’ digital leadership, employee empowerment, affective commitment, and work engagement

According to Social Exchange Theory, when middle managers support their employees, reciprocal exchange relationships are initiated, compelling employees to act in response to the managers’ support. Middle managers’ digital leadership, through digital technology, facilitates employee empowerment. This empowerment enhances employees’ work capabilities during digital transformation and their representation within the organizational hierarchy, which in turn helps guide their identification with organizational values and subsequently fosters affective commitment to the organization (Brunetto et al., 2012) Furthermore, middle managers’ digital leadership equips employees with high-quality information and necessary resources during digital transformation, along with increased support, learning, and growth opportunities, fueling their attachment to the organization (Prentice et al., 2020). Therefore, this study contends that employee empowerment has a significant positive effect on organizational commitment. Combined with the above hypotheses, this study posits that middle managers’ digital leadership can enhance employees’ affective commitment levels through employee empowerment, which in turn increases work engagement. Based on this rationale, the following hypothesis is proposed:

*H4*: Employee empowerment and affective commitment play a chain mediating role in the relationship between middle managers’ digital leadership and employee work engagement.

### 3.5 The moderating role of emotional intelligence

During an enterprise’s digital transformation, employees often encounter strong emotional reactions due to factors like overwhelming workloads, unrealistic deadlines, time pressures, and a lack of leadership support (Mayer et al., 2008). The digital office environment, without body language and interpersonal cues, further complicates the task of perceiving and identifying emotions. Employees are not only required to accurately understand their own and others’ emotions but also to respond appropriately, promoting adaptive social behaviors. This capability demands a high level of emotional intelligence. Emotional intelligence encompasses the ability to perceive, respond to, and manage emotional information, and understand and govern emotions, forming part of social intelligence (Alotaibi et al., 2020). It includes the skills of emotional perception, assimilation, understanding, and management skills. Emotional perception and assimilation are foundational for accurately understanding and managing emotions. In interactions with middle managers, employees with lower levels of emotional intelligence may not fully grasp the developmental information provided by the managers. In contrast, those with higher emotional intelligence levels not only comprehend the intentions of their managers but also swiftly assimilate and integrate the quality information resources offered (Roman et al., 2019). Additionally, employees with higher emotional intelligence typically possess robust interpersonal networks within the organization, holding more social capital and work resources, and exerting greater influence and control (Zhu et al., 2015). Consequently, employees with higher emotional intelligence perceive greater empowerment under middle managers’ digital leadership. Conversely, those with lower emotional intelligence levels may not fully understand the managers’ intentions or grasp key resource information, leading to reduced empowerment perception. Considering hypotheses H1, H2, H3, and H4, this study posits that the interaction between middle managers’ digital leadership and employees’ emotional intelligence impacts work engagement through its influence on employee empowerment perception and affective commitment. In light of this, the study proposes a moderated mediation hypothesis:

*H5*: Emotional intelligence moderates the influence of middle managers’ digital leadership on employee empowerment, subsequently affecting affective commitment and e work engagement.

**Figure 1:**
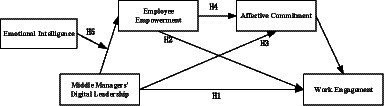
Research model.

Based on the presented analysis, the research model for this study is illustrated in Figure 1.

## 4 Methods

### 4.1 Procedures and samples

The respondents of this study are mainly from 11 publicly listed companies in the southwest region of China undergoing digital transformation. These companies are involved in various industries, including automotive manufacturing, white goods manufacturing, chemical and pharmaceuticals, food processing, and construction. Compared to small and medium-sized enterprises, listed companies have more economic resources to invest in and carry out digital transformation. Before distributing the questionnaires, the middle management and their corresponding employees were numbered with the assistance of the company’s human resource managers. Based on these numbers, questionnaires were distributed to the respondents. For on-site employees, a questionnaire survey box was set up in each company, and questionnaires were distributed during work-free hours. Respondents completed and directly deposited their questionnaires into the box. For employees who were in business traveling or expatriate staff, electronic survey links were sent to their email addresses with the help of human resource management personnel, instructing them to fill out the online questionnaire.

To minimize the impact of common method bias on the study’s results, a two-stage data collection method was employed, with a one-month interval between the stages. At time point one, employee demographic information, middle managers’ digital leadership levels (rated by employees on their corresponding middle managers), and information on employees’ affective commitment were collected. At time point two, data on employees’ emotional intelligence, empowerment perception, and work engagement were gathered. A total of 840 questionnaires were distributed at time point one, with 697 complete questionnaires returned, resulting in a response rate of 82.97%. At time point two, questionnaires were sent to those who completed the first survey, yielding 634 complete questionnaires and a response rate of 93.37%. After excluding 75 invalid questionnaires, a total of 559 effective questionnaires were obtained, with an effective response rate of 66.54%. Descriptive statistics revealed that 47.9% of the respondents were female, 53.7% fell within the age bracket of 21–30, and a significant majority, totaling 73%, held at least a bachelor’s degree.

### 4.2 Measures

In this research, we employed rigorously vetted scales from both national and international sources. Each scale adopts a 5-point Likert scoring system, ranging from “1” (strongly disagree) to “5” (strongly agree), to measure various aspects of organizational behavior and employee perceptions.

#### 4.2.1 Middle managers’ digital leadership

Utilizing an 18-item scale developed by Roman et al. (2019), this aspect assesses the effectiveness of middle managers in fostering digital transformation within teams. For example, one item reads, “My middle manager has successfully established a high-efficiency digital workforce during the digital transformation phase.” The reliability of this scale, indicated by a Cronbach’s alpha coefficient, is 0.919.

#### 4.2.2 Emotional intelligence

Measured using a 16-item scale by Law et al. (2004), this scale probes into the respondents’ self-awareness of their emotional triggers and reaction. One representative item is, “Generally, I am aware of the reasons behind my varied emotional states.” The scale demonstrates strong internal consistency with a Cronbach’s alpha of 0.909.

#### 4.2.3 Employee empowerment

Drawing on the foundational work of Rogers et al. (1997), and contextualized for the Chinese cultural setting, this 21-item scale assesses the degree of empowerment or disempowerment employees feel in their roles. An illustrative item is, “I am confident in the effectiveness of the work plans I have made.” This scale is characterized by high reliability, with a Cronbach’s alpha of 0.915.

#### 4.2.4 Affective commitment

The scale is based on a 9-item scale designed by Tsui et al. (1997), to reflect the emotional bond and commitment employees feel toward their organization. For instance, one of the items states, “I am committed to going above and beyond for the success of the organization.” The scale’s reliability is reflected in its Cronbach’s alpha of 0.925.

#### 4.2.5 Work engagement

Following a 9-item scale designed by Schaufeli et al. (2006), this scale evaluates the level of energy and engagement employees experience in their work. An example of an item from this scale is, “I feel energized in my position.” The scale demonstrates a Cronbach’s alpha of 0.883, indicating good reliability.

#### 4.2.6 Control variables

Control variables at the individual and organizational levels were carefully selected based on previous research and the specifics of this study. At the individual level, variables such as gender, age, and educational background were included. At the organizational level, the nature of the enterprise was incorporated as a control variable. This dual-level approach ensures a comprehensive understanding of the factors influencing the study’s outcomes.

## 5 Results

### 5.1 Common method bias, confirmatory factor analysis, and descriptive statistics

To address potential issues of common method bias, this study initially employed Harman’s single-factor test. The data for five variables – middle managers’ digital leadership, emotional intelligence, employee empowerment, affective commitment, and work engagement – were subjected to a principal component analysis without rotation. The results revealed that the total variance explained amounted to 66.928%, of which the principal factor accounted for 25.904% of the variance, which is less than 40% and below half of the total variance explained. These findings suggest that the common method bias in this study is within an acceptable range.

Furthermore, the study utilized Mplus 8.3 software to conduct a confirmatory factor analysis of the five variables. As shown in Table 1, the five-factor model achieved a more satisfactory level of fit compared to other models, demonstrating that the constructs in this study have good discriminant validity. This analysis underscores the distinctiveness and individual contributions of each construct within the research framework.

**Table 1:** Results of confirmatory factor analysis.

Model	*X*^2^/DF	GFI	NFI	TLI	CFI	RMSEA
One factor model DL + EE + AC + WE+EI	8.099	0.649	0.668	0.669	0.696	0.113
Two factors model DL + EE + AC + EI,WE	7.493	0.666	0.694	0.697	0.722	0.108
Three factors model DL,EE + AC + EI,WE	5.173	0.774	0.790	0.805	0.823	0.086
Four factors model DL,EE + AC,EI,WE	3.304	0.855	0.867	0.893	0.903	0.064
Five factors model DL,EE,AC,EI,WE	1.524	0.943	0.940	0.976	0.978	0.031

Digital Leadership, DL; Work Engagement, WE; Employee Empowerment, EE; Affective Commitment, AC; Emotional Intelligence, EI.

As shown in Table 2, the mean values, standard deviations, correlation coefficients, and significance levels of each variable do not show any abnormal values. The results indicate a significant positive correlation between middle managers’ digital leadership level and employee empowerment perception, affective commitment, and employee work engagement. Additionally, emotional intelligence is positively correlated with both middle managers’ digital leadership level and employee empowerment perception. These findings lay a solid foundation for subsequent hypothesis testing.

**Table 2:** Results of descriptive statistical analysis.

Variable	M	SD	DL	EE	AV	EI
DL	3.9799	0.55198				
EE	4.0175	0.51116	0.510**			
AC	3.8897	0.70137	0.483**	0.520**		
EI	3.9653	0.53146	0.352**	0.339**	0.286**	
WE	3.9447	0.61245	0.429**	0.497**	0.543**	0.325**

Digital Leadership, DL; Work Engagement, WE; Employee Empowerment, EE; Affective Commitment, AC; Emotional Intelligence, EI; **p < 0.01; *p < 0.05.

### 5.2 Hypothesis testing

To enhance commonality and reduce the impact of random errors on the research outcomes, this study employed the internal consistency method for item parceling. Subsequently, hypothesis testing was conducted using Mplus 8.3 software through structural equation modeling. Initially, the main effect of middle managers’ digital leadership level on employee work engagement was tested. The main effect structural equation model exhibited good fit (*X*^2^/DF = 1.225, GFI = 0.988, NFI = 0.983, TLI = 0.996, CFI = 0.997, RMSEA = 0.037). The standardized path coefficient is positive and significant (*β* = 0.657, *p* < 0.001), indicating that middle managers’ digital leadership level significantly positively influences employee work engagement, thus supporting Hypothesis H1.

**Figure 2:**
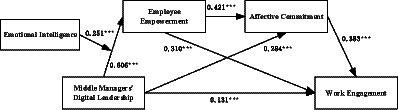
Estimates of the moderated-mediation model. ***p < 0.001.

Next, considering the presence of three types of multiple mediation models - pure chain mediation, parallel mediation, and compound mediation - this study initially constructed a chained multiple mediation Model A. In this model, middle managers’ digital leadership level was the independent variable, employee work engagement was the dependent variable, and employee empowerment perception and affective commitment were the mediators, as depicted in Figure 2. Based on Model A, the paths from middle managers’ leadership level to affective commitment and from employee empowerment perception to work engagement were removed to transform into a pure chain mediation Model B. In addition, removing the path from employee empowerment perception to affective commitment on Model A’s basis transformed it into a parallel mediation Model C. The results, as shown in Table 3, indicated that Model A had the best fit compared to the other models. When Δ*X*^2^ is significantly different, the more complex model with better fit is considered optimal. As shown in Table 3, Model A, compared to Models B and C, showed significant changes in Chi-square value (Δ*X*^2^(B) = 47.407, *p* < 0.01; Δ*X*^2^(C) = 56.758, *p* < 0.01), suggesting that Model A is the optimal model. Hence, middle managers’ digital leadership level not only improves employee work engagement through employee empowerment and affective commitment independently but also influences work engagement by first enhancing employee empowerment perception, which then fosters affective commitment.

**Table 3:** Fit indices of structural equation model.

Model	*X* ^2^	DF	*X*^2^/DF	GFI	NFI	TLI	CFI	RMSEA
Model A	358.216	203	1.765	0.945	0.945	0.972	0.975	0.037
Model B	405.623	205	1.979	0.938	0.937	0.964	0.968	0.059
Model C	414.947	204	2.034	0.937	0.936	0.962	0.966	0.043

Finally, based on the structural equation model and using the Bootstrap method with 5,000 iterations and a 95% confidence interval, we tested the chain multiple mediation effects of employee empowerment perception and affective commitment. The optimal mediation model and Bootstrap test results are illustrated in Figure 2. After controlling for variables such as gender, age, education, and length of service, the following results were obtained:

The path coefficient from middle manager digital leadership to employee empowerment perception (*β* = 0.606, *p* < 0.001) and from employee empowerment perception to work engagement (*β* = 0.31, *p* < 0.001) are significant. The mediating effect of employee empowerment perception between middle manager digital leadership level and employee work engagement is significant (*β* = 0.188, *p* < 0.001), with a confidence interval of [0.111, 0.265] that does not include 0, supporting Hypothesis H2.

The path coefficient from middle manager digital leadership to employee affective commitment (*β* = 0.284, *p* < 0.001) and from employee affective commitment to work engagement (*β* = 0.383, *p* < 0.001) are both significant. The mediating effect of employee affective commitment between middle manager digital leadership level and employee work engagement is significant (*β* = 0.109, *p* < 0.001), with a confidence interval of [0.070, 0.154] that does not include 0, supporting Hypothesis H3.

The path coefficient from employee empowerment perception to affective commitment is significant (*β* = 0.421, *p* < 0.001). The chain mediation effect of employee empowerment perception and affective commitment between middle manager digital leadership and employee work engagement is significant, with a confidence interval of [0.069, 0.135] that does not include 0, supporting Hypothesis H4. Simultaneously, the path coefficient from middle manager digital leadership to employee work engagement is significant (*β* = 0.131, *p* < 0.05), with a direct effect confidence interval of [0.025, 0.230] that does not include 0, indicating that employee empowerment perception and affective commitment partially mediate between middle manager digital leadership and employee work engagement (Table 4).

**Table 4:** Bootstrap mediation effect estimates and 95% confidence interval.

Pathway	Indirect Effect (Standardized)	95% Confidence Interval	
		Upper	Lower
Total Indirect Effect	0.395***	0.453	0.592
DL → EE → WE	0.188***	0.111	0.265
DL → AC → WE	0.109***	0.070	0.154
DL → EE → AC → WE	0.098***	0.069	0.135

Digital Leadership, DL; Work Engagement, WE; Employee Empowerment, EE; Affective Commitment, AC; Emotional Intelligence, EI; ***p < 0.001; **p < 0.01; *p < 0.05.

**Figure 3:**
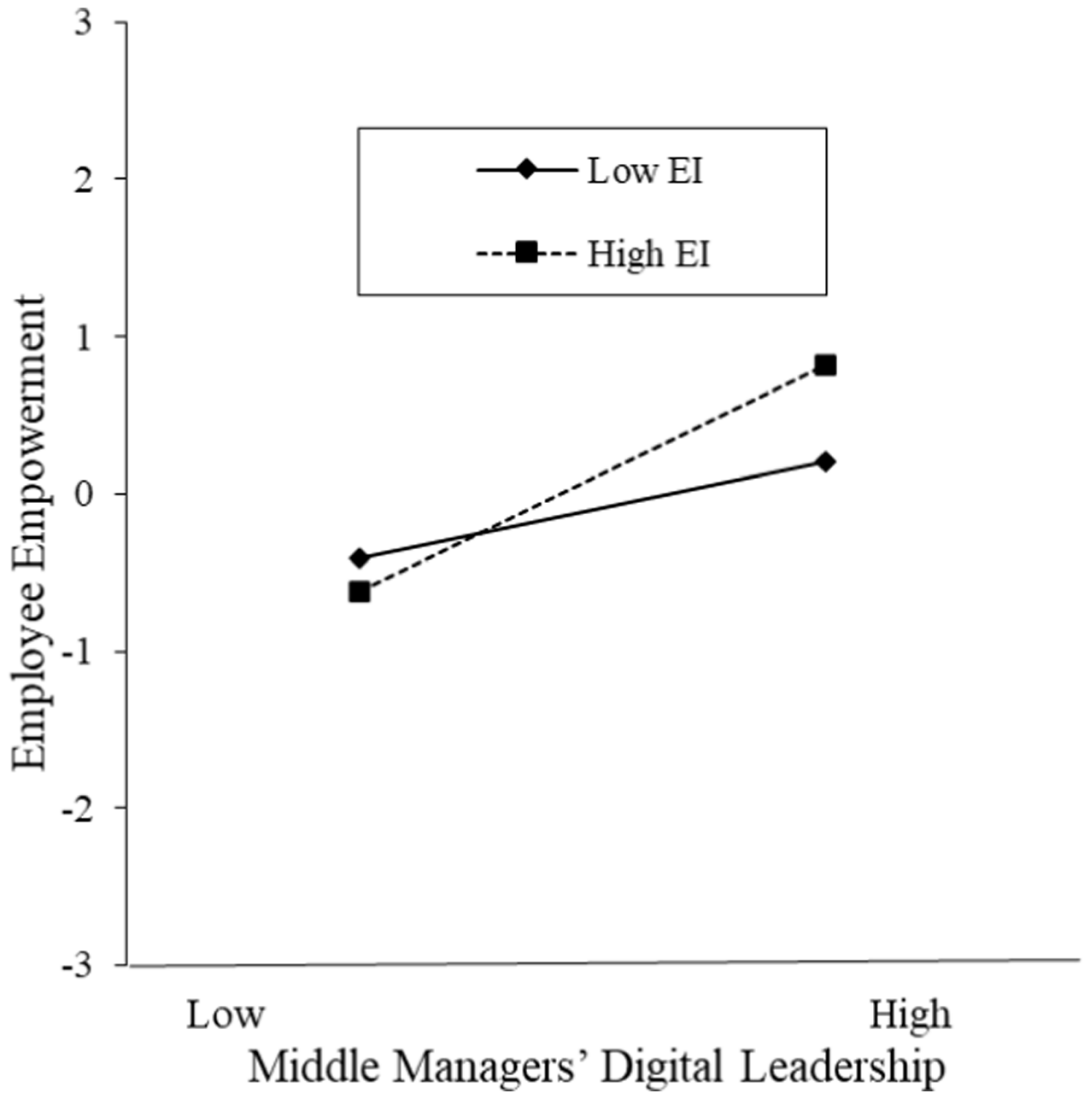
Analysis of the moderating effect.

This study employs Latent Moderated Structural Equations (LMS) methodology to test the moderated-mediation model algorithm. The results reveal a significant interaction effect between middle managers’ digital leadership and employee emotional intelligence on employee empowerment (*β* = 0.251, *p* < 0.001). This finding indicates that employee emotional intelligence moderates the relationship between middle managers’ digital leadership and employees’ perception of empowerment. As illustrated in Figure 3, when employees exhibit higher levels of emotional intelligence, the positive impact of middle managers’ digital leadership on employee empowerment perception is significant (*β* = 0.720, *t* = 10.675, *p* < 0.001). Conversely, at lower levels of emotional intelligence, the positive influence of middle managers’ digital leadership on employee empowerment perception remains significant (*β* = 0.302, *t* = 4.480, *p* < 0.001). These results demonstrate that employee emotional intelligence has a significant moderating effect on middle managers’ digital leadership on employee empowerment perception.

To test the moderated chain mediation effect, this study employed the coefficient product proposed by Igartua and Hayes (2021) to assess the significance of the moderated mediation model. Additionally, the study used the differential analysis suggested by Edwards and Lambert (2007) to further validate the significance of the differences in the mediation effects. The results indicated that in the chain mediation effect of employee empowerment and affective commitment between middle managers’ digital leadership and employee work engagement, the product of the path coefficients between the interaction term and the mediating variables was significant at 0.125 (*p* < 0.001), demonstrating that the chain mediation effect was moderated by emotional intelligence. As shown in Table 5, when the level of employees’ emotional intelligence was low (one standard deviation below the mean), the value of the chain mediation effect was 0.063 (*p* < 0.001), with a confidence interval of [0.039, 0.100]. When the level of emotional intelligence was high (one standard deviation above the mean), the value of the chain mediation effect was 0.148 (*p* < 0.001), with a confidence interval of [0.103, 0.205]. Moreover, there was a significant difference in the chain mediation effect between high and low levels of emotional intelligence (*p* < 0.001, CI [0.053, 0.127]), indicating that the chain mediation effect of employee empowerment and affective commitment between middle managers’ digital leadership and employee work engagement was significantly stronger at higher levels of emotional intelligence compared to lower levels. Consequently, Hypothesis H5 was supported.

**Table 5:** Moderated chain mediation.

Moderating Effect	Pathway:DL → EE → AC → WE		
	Indirect Effect	95% Confidence Interval	
		Lower	Upper
Low EI	0.063***	0.039	0.100
High EI	0.148***	0.103	0.205
Difference	0.085***	0.053	0.127

Digital Leadership, DL; Work Engagement, WE; Employee Empowerment, EE; Affective Commitment, AC; Emotional Intelligence, EI. ***p < 0.001.

## 6 Discussion

### 6.1 Key conclusion

While extensive research has been conducted on digital leadership based on higher-order theories, primarily focusing on its concepts, characteristics, capability building, and its role in corporate digital transformation, there has been relatively little research on the impact of digital leadership within the organization, particularly the influence of middle managers as pivotal figures in digital transformation, has been relatively underexplored. This study, based on Social Exchange Theory, investigates the effect of middle managers’ digital leadership on employee work engagement. The findings reveal several critical insights: Middle managers’ digital leadership significantly and positively influences employee work engagement. Employee empowerment mediates the positive relationship between middle managers’ digital leadership and employee work engagement. Employee affective commitment also serves as a mediate factor, indicating that middle managers’ digital leadership positively impacts employee work engagement through affective commitment. A chain mediating role of both employee empowerment and affective commitment exists between middle managers’ digital leadership and employee work engagement. The study uniquely demonstrates that employee emotional intelligence moderates the chain-mediated pathway of “middle managers’ digital leadership - employee empowerment - affective commitment - work engagement.” Specifically, higher levels of employee emotional intelligence significantly enhance the chain mediation effect compared to lower levels. Overall, this research contributes to the understanding of digital leadership in a corporate setting, emphasizing the critical role of middle managers and how their digital leadership approach can effectively foster employee work engagement in the context of digital transformation.

### 6.2 Theoretical contributions

Grounded in Social Exchange Theory, this study embarks on a critical examination of middle managers’ digital leadership and its influence on employee work engagement, offering substantial contributions to the discourse on middle management leadership. As organizations pivot toward decentralized, network-oriented structures catalyzed by digital technology, the nuanced role of middle managers in this digital transformation landscape becomes increasingly pertinent. While existing literature has explored the dynamics of middle managers in digital transformation, notably their interaction with top-level management, there remains a critical gap in understanding their role in fostering employee engagement in digital contexts. Our research addresses this oversight by scrutinizing the exchange dynamics between middle managers and employees, highlighting the significant impact of middle managers’ digital leadership in shaping employee perceptions and engagement.

This research focuses on the influence of middle managers’ digital leadership on employee work engagement. By exploring the mechanisms through which middle managers’ digital leadership impacts employee work engagement, this study validates the positive effects of digital leadership on employees’ work-related behaviors and attitudes. While existing research on digital leadership primarily revolves around its impact on organizational outcomes, there has been limited exploration of its effects on individual employees. Additionally, most of these studies have been rooted in high-level theoretical perspectives, often neglecting the specific role of middle managers’ digital leadership. Set against the backdrop of corporate digital transformation, this research confirms the influence of middle managers’ digital leadership on employee work engagement. Our findings reveal that middle managers’ digital leadership not only directly affects employee work engagement but also indirectly influences it by enhancing employee empowerment and affective commitment. Specifically, higher levels of middle managers’ digital leadership are associated with stronger perceptions of empowerment among employees, leading to increased affective commitment toward middle managers and, subsequently, more proactive work engagement. Therefore, this study significantly supplements existing research on the impact of middle managers’ digital leadership on employee perceptions and behaviors, offering new insights into the dynamics of digital leadership within organizational settings. Our investigation into the mechanisms of middle managers’ digital leadership on employee work engagement illuminates the positive effects of digital leadership on individual employee attitudes and behaviors, a facet previously underexplored in digital leadership studies. The majority of existing research has emphasized the organizational-level impacts of digital leadership, often neglecting its influence on individual employee experiences. Moreover, the distinction between senior and middle managerial digital leadership and their respective influences has been largely overlooked. This study bridges these gaps, focusing on the multifaceted roles of middle managers in the era of digital transformation and elucidating the pathways through which their digital leadership enhances employee work engagement. This nuanced approach significantly enriches the digital leadership narrative by providing deeper insights into its effects across various managerial levels.

Furthermore, this study introduces emotional intelligence as a key moderating variable and employs the Latent Moderated Structural Equations (LMS) model to examine the differential impact of middle managers’ digital leadership on employee work engagement under varying levels of emotional intelligence. While the application of digital technologies is crucial for employees to adapt to digital transformation, emotional intelligence plays a vital role in helping employees better understand and manage their emotional reactions in a rapidly changing digital environment. From the perspective of Social Exchange Theory, this research elucidates the role of emotional intelligence in enhancing the effectiveness of middle managers’ digital leadership and its impact on employee work engagement. Emotional intelligence enhances employees’ ability to effectively interact with middle managers in the digital transformation process, which involves not only technical adaptation but also emotional and social exchanges. Higher emotional intelligence allows employees to better comprehend and respond to the leadership behaviors of middle managers, leading to a more positive response in the social exchange process. This enhanced social exchange relationship fosters increased perceptions of employee empowerment and affective commitment among employees, ultimately resulting in higher levels of work engagement. Therefore, this study not only enriches the application of Social Exchange Theory in the field of digital leadership research but also provides a new perspective in understanding the mechanisms of how emotional intelligence influences employee behavior in the context of digital transformation. Through this comprehensive analysis, we gain a deeper understanding of how to enhance employee participation and work engagement in a digital environment by strengthening the digital leadership capabilities of middle managers.

### 6.3 Practical implications

In the realm of organizational digital restructuring, it’s critical to conduct a thorough assessment and ensure the preservation of middle managers’ roles. Companies must adopt strategic and actionable steps to navigate this transformation. Foremost, clearly defining middle managers’ roles and responsibilities is essential, making them aware of their significant impact on organizational change. The creation of a specialized digital leadership program, which includes tailored training sessions, workshops, and online modules, is crucial for enhancing their digital skills and strategic acumen.

Furthermore, offering middle managers the chance to actively contribute to the design and implementation of digital strategies enriches their understanding and commitment. Promoting cross-functional collaboration is key to a unified digital transformation effort across the organization, preventing the emergence of informational silos. It’s imperative for senior management to exhibit trust and respect toward middle managers by sharing decision-making power and providing necessary resources, such as time and financial backing, to encourage their active involvement in shaping digital strategies. Continuous evaluation and feedback are vital in supporting middle managers’ growth and progress in the digital transformation journey, facilitating the organization’s successful digital evolution.

For middle managers to effectively merge digital technology with leadership and aid the organization’s digital transformation, a structured approach is necessary. Organizations should offer specialized training that updates managers on the latest digital advancements and their application in leadership roles. Middle managers are urged to cultivate a digital mindset by participating in digital initiatives and leading transformation projects, adapting to the technological changes within the organization. They also need to improve their digital communication capabilities, leveraging online platforms to reduce misinformation and boost communication efficiency. Establishing a central platform for information sharing can enhance knowledge exchange, teamwork, and expedite decision-making processes. Middle managers should employ digital tools for self-disclosure, fostering trust and security, while also motivating employees to share feedback and seek support via digital channels, thus enhancing engagement and collaboration. Implementing these strategies will fortify the digital leadership of middle managers, pivotal for steering the organization through the digital era and boosting employee engagement.

Employees play a crucial role in the digital transformation process, particularly in decision-making and resource integration within a digital workspace. They should grasp opportunities to engage in decision-making, honing their abilities to recognize, comprehend, and apply emotional intelligence in a digital context. Employees should leverage the organizational environment and resources to continually advance their learning, application, and innovation capabilities in digital technologies, keeping pace with technological advancements. Employee empowerment relies on establishing trust with managers and active participation and collaboration among employees. Thus, employees should actively engage in the company’s digital transformation efforts, enhancing their organizational impact through active participation in meetings and decision processes, gaining access to more personal and professional resources. This proactive engagement not only strengthens the dynamic between employees and the organization but also contributes to the success of digital transformation initiatives, increasing employees’ sense of professional achievement and fulfillment.

### 6.4 Limitations and future directions

This study, while methodologically sound, has its limitations. First, despite employing a two-phase research design at different time points to mitigate common method bias, the reliance on self-reported data from employees could not entirely eliminate the potential adverse effects of this bias. Future research could enhance the study design by integrating both subjective and objective data collection methods. Second, the companies investigated in this study were primarily located in the Southwest China. Given the diversity in industries and geographical areas, this might have introduced sampling errors, limiting the generalizability of our findings. Future studies could address this limitation by including a more diverse sample that spans different regions and industries. Finally, this research explored the multiple mediating mechanisms of middle managers’ digital leadership on employee work engagement through the lens of Social Exchange Theory. However, it did not examine the boundary conditions under which digital leadership affects employee work engagement. Future research could build on this study by investigating these conditions, thereby providing a more comprehensive understanding of the impact of digital leadership in organizational contexts.

## Data availability statement

The raw data supporting the conclusions of this article will be made available by the authors, without undue reservation.

## Author contributions

ZL: Conceptualization, Data curation, Investigation, Methodology, Software, Supervision, Writing – original draft, Writing – review & editing. CY: Writing – review & editing. ZY: Writing – review & editing. YZ: Resources, Writing – review & editing.
