# Supplementary material for: The impact of middle managers’ digital leadership on employee work engagement
Source: Front Psychol. 2024 Mar 28;15:1368442. doi: 10.3389/fpsyg.2024.1368442 (PMC11008576; doi:10.3389/fpsyg.2024.1368442)
Supplement: Supplementary file 1 [file Table_1.docx]

**Supplementary Table 1: Results of Confirmatory Factor Analysis**

| **Variables** | **Items** | **Factor loading** |
| --- | --- | --- |
| **Middle Managers' Digital Leadership (DL)** | My middle manager is logical and well organized in language during digital communication and allows subordinates to give feedback. | 0.682 |
|  | My middle manager sent inaccurate messages that led me to misunderstand and even feel insulted during digital communication. | 0.640 |
|  | I don't receive too many messages from My middle manager and it doesn't get in the way of normal work during digital communication. | 0.678 |
|  | My middle manager provides personalized digital communication for employees. | 0.633 |
|  | My middle manager uses a variety of digital communication methods. | 0.684 |
|  | My middle manager used the digital platform to promote the communication and collaboration of the team. | 0.657 |
|  | My middle managership built an efficient digital work team. | 0.652 |
|  | My middle manager can effectively motivate members of the digital work team. | 0.625 |
|  | My middle manager is responsible for the work of the digital work team. | 0.648 |
|  | My middle managers can effectively use digital platforms to plan corporate changes. | 0.608 |
|  | My middle manager can monitor the changes of the enterprise through the digital platform. | 0.564 |
|  | My middle managers can accurately evaluate enterprise change plans through digital platforms. | 0.596 |
|  | My middle managers kept abreast of the technologies and new functions of various digital platforms. | 0.547 |
|  | My middle managers have sufficient skills to deal with the failures of digital platforms. | 0.609 |
|  | My middle manager is very knowledgeable about network security. | 0.604 |
|  | I fully trust My middle manager in the digital setting. | 0.637 |
|  | My middle managers are very honest and trustworthy in the digital platform, and they are fair and consistent. | 0.599 |
|  | My middle managers provide adequate support and supervision for employees in the digital platform. | 0.571 |
| **Employee Empowerment**  **(EE)** | When I make a work plan, I can be sure that it will work. | 0.673 |
|  | I feel I have the ability to do well in all aspects of my job. | 0.647 |
|  | I have great confidence in my ability to get the job done. | 0.756 |
|  | I'm confident in the decisions I've made at work. | 0.721 |
|  | I can overcome all kinds of obstacles in my work. | 0.602 |
|  | I often don't feel overwhelmed by my work. | 0.574 |
|  | When I'm not sure about my job, I usually stick to my own ideas. | 0.650 |
|  | I often don't feel lonely in this company. | 0.562 |
|  | I can decide for myself how to do my job, even if I make bad decisions. | 0.630 |
|  | I have strong independence and autonomy in how to accomplish my work. | 0.596 |
|  | Working with colleagues can have a strong impact on the company. | 0.601 |
|  | When colleagues are united, they have more power. | 0.588 |
|  | Working with my colleagues helps me work more smoothly. | 0.617 |
|  | In the workplace, I am only influenced and limited by my own concerns. | 0.666 |
|  | I can determine the direction and content of my future work. | 0.626 |
|  | When I encounter problems in my work, I can solve them by acting accordingly. | 0.654 |
| **Work Engagement**  **(WE)** | When I get up in the morning, I feel I like going to work. | 0.703 |
|  | At my job, I feel strong and vigorous. | 0.816 |
|  | At my work, I fell bursting with energy. | 0.722 |
|  | I am enthusiastic about my job. | 0.697 |
|  | My job inspires me. | 0.682 |
|  | I proud of the work that I do. | 0.734 |
|  | I feel happy when I am working intensely. | 0.604 |
|  | I am immersed in my work. | 0.540 |
|  | I get carried away when I am work. | 0.598 |
| **Affective Commitment**  **(AC)** | I am willing to put in effort beyond the norm for the success of the organization. | 0.673 |
|  | I talk up this organization to my friends as a great place to work for. | 0.766 |
|  | I find that my values and the organization's values are very similar. | 0.770 |
|  | I am proud to tell others that I am part of this organization. | 0.786 |
|  | This organization inspires the very best in the way of job performance. | 0.794 |
|  | I am extremely glad to have chosen this organization to work for over other organizations. | 0.797 |
|  | I would accept almost any type of job to keep working for this organization. | 0.810 |
|  | I really care about the fate of this organization. | 0.671 |
|  | For me this is the best of all possible organizations for which to work. | 0.792 |
| **Emotional Intelligence**  **(EI)** | I have a good sense of why I have certain feelings most of the time. | 0.576 |
|  | I have good understanding of my own emotions. | 0.601 |
|  | I really understand what I feel. | 0.661 |
|  | I always know whether or not I am happy. | 0.533 |
|  | I always know my friends’ emotions from their behavior | 0.594 |
|  | I am a good observer of others’ emotions. | 0.632 |
|  | I am sensitive to the feelings and emotions of others. | 0.549 |
|  | I have good understanding of the emotions of people around me. | 0.610 |
|  | I always set goals for myself and then try my best to achieve them. | 0.698 |
|  | I always tell myself I am a competent person | 0.673 |
|  | I am a self-motivating person. | 0.689 |
|  | I would always encourage myself to try my best | 0.659 |
|  | I am able to control my temper so that I can handle difficulties rationally. | 0.617 |
|  | I am quite capable of controlling my own emotions. | 0.621 |
|  | I can always calm down quickly when I am very angry. | 0.640 |
|  | I have good control of my own emotions. | 0.617 |
